# Impact of unilateral removable partial dentures versus removable partial dentures with major connector on oral health-related quality of life of elder patients: a clinical study

**DOI:** 10.1186/s12903-023-02870-x

**Published:** 2023-03-29

**Authors:** Luciana Goguta, Mirela Frandes, Adrian Candea, Codruta Ille, Anca Jivanescu

**Affiliations:** 1 Department of Prosthodontics, Faculty of Dentistry, “Victor Babes” University of Medicine and Pharmacy, Timisoara, Romania; 2 Department of Functional Sciences - Biostatistics and Medical Informatics, Faculty of Medicine, “Victor Babes” University of Medicine and Pharmacy, Timisoara, Romania

**Keywords:** Patient satisfaction, Removable partial dentures, Oral health, Quality of life, Survival analysis

## Abstract

**Objective:**

This study aimed to establish the survival rate of unilateral removable partial dentures (u-RPD) comparative with bilateral RPD (bi-RPD) with major connector in elder patients, as well as to determine both their treatment satisfaction and oral health.

**Methods:**

The study sample included 17 patients treated with u-RPD and 17 patients treated with bi-RPD with a major connector. The patients were followed over five years with recalls every 6 months. A 5- points Likert scale was used to determine the satisfaction of the patients. The Oral Health Impact Profile-14 (OHIP-14) questionnaire was used to evaluate their oral health after each type of administrated treatment. The local oral examined aspects included the maintenance of the abutment teeth periodontal health, the fractures of the removable dentures, the fractures of the connectors, the chipping of the aesthetic material. Kaplan–Meier survival analysis was conducted to evaluate the performance of the two treatments.

**Results:**

The mean survival time in years was 4.882 ± 0.114, 95% CI (4.659; 5.106) and 4.882 ± 0.078, 95% CI (4.729; 5.036), for the u-RPD and the bi-RPD, respectively. The five-year survival rates for the two dentures were 94.1% for u-RPD vs. 88.2% for bi-RPD with a major connector, without a statistically significant difference between them (Log-rank test χ2(1) = 0.301, p = 0.584). The patients receiving u-RPD presented significantly higher satisfaction scores compared to the patients receiving bi-RPD, 4.88 ± 0.48 vs. 4.41 ± 0.62, Mann-Whitney U test, p = 0.026.

**Conclusion:**

Patients receiving u-RPD presented higher levels of treatment satisfaction and better oral health than patients receiving bi-RPD. The survival rates of the treatments u-RPD and bi-RPD were similar.

## Background

Due to an increasing aging population retaining more teeth at an older age, in the era of fixed partial dentures, the removable partial dentures (RPD) are still used to restore edentate patients. Psychological factors are important in patients suffering from tooth loss and/or in those awaiting prosthetic care with fixed or removable dentures [1, 2]. Also, it was demonstrated that denture satisfaction is the strongest predictor of oral health-related quality of life (OHRQoL) [3].

It would be correct to state that removable dental prostheses, given suitable pretreatment and follow-up regimes, can provide satisfactory solutions [4]. It was suggested that RPDs improve mastication in extremely shortened dental arches subjects, but without achieving normal mastication levels [5]. Also, it was shown that the ability of chewing and the OHRQoL status in patients with partially dentition are significantly related and influenced by denture status and nonclinical characteristics [6].

Conventional RPD design is frequently bilateral [7] and consists of a major connector that bridges both sides of the arch. However, some patients cannot and will not tolerate such an extensive appliance. For these patients, fixed partial denture (FPD) may not be a predictable option and it is not always possible to provide implant-retained restorations [8]. In these cases, the patients are preferring to realize the mastication using only the remaining natural teeth, instead of wearing and using the removable denture with major connector. There are many patients who are not even aware of the importance of hygiene and maintenance of the removable dentures [9, 10].

Studies have suggested that at least two teeth on each side should be splinted when extra-coronal distal extension attachment prostheses are used [11, 12]. It is important to protect the periodontal health of the abutment when restored with distal-extension extra-coronal [13]. Stress on the terminal abutment can be reduced by using an extra - coronal resilient attachment that allocates more loads onto the distal edentulous ridge [14–16].

The new fabrications techniques for RPD like subtractive CAD-CAM (computer aided design – computer aided manufacturing) or additive SLM (selective laser sintering) and SLS (selective laser melting) are offering promising results [17–20].

The RDP retention includes the selection of the potential abutment teeth taking into the account their prognosis, their position in the arch, as well as the planned prosthesis design. Retainer selection mainly depends on the remaining tooth substance, the intra- and inter-maxillary relationships, esthetics, and financial aspects. The benefits of dental implants as additional retainers are the increased supportive area for the RDP, the minimized load of the soft tissue, as well as the reduced extension of the base of the prosthesis to enhance the patient’s comfort [21].

The aim of this clinical study was to establish the survival of unilateral RPD (u-RPD) compared to bilateral RPD (bi-RPD) with major connector, to assess the overall satisfaction of the patients wearing these dentures as well as their OHRQoL.

## Methods

### Study design and patients

The study was realized in the Department of Prosthodontics, Faculty of Dentistry, “Victor Babes” University of Medicine and Pharmacy, Timisoara, Romania during 2016–2021. Thirty-four partial edentate patients were included in the study. Each patient has signed an informed consent in accordance with an Agreement from the Ethical Committee of the University. The study was conducted according to the Declaration of Helsinki.

17 patients were treated with u-RPD, and a group of patients with the same size (17) were treated with bi-RPD with a major connector (control group). Both type of dentures had a metal frame and precision attachment: Rhein 83 OT unilateral (Italy) and The F.M. hinge (New Ancorvis, Italy), for the u-RPD and external slide attachments, Vario-Soft 3 rod attachment (Bredent, Germany), for the bi-RPD (control group). In the two cases with extended class Kennedy III edentulous areas, on the mesial abutments it was used a resilient (Rhein 83 OT unilateral Italy) and on the distal abutment a telescopic crown (coping and telescopic crown relined with resilient material FGP (Bredent, Germany).

The patient’s inclusion criteria were: (1) patients without neurological diseases; (2) presenting Kennedy class I, II or extended class III edentate arches, with or without modifications, incorrect treated or not treated; 2. abutment teeth with healthy periodontium or incipient periodontitis (3) the impossibility of realizing implant supported FPD; (4) recalls every six months; (5) direct reline every 12 months? The exclusion criteria were: (1) missing the recalls; (2) missing the reline appointments. (3) patients who do not wear their removable dentures. Following these criteria, the patients were randomly selected.

The examined aspects were (a) maintenance of the occlusal contacts and the masticatory function; (b) maintenance of the periodontal health of the abutment teeth; (c) fractures of the removable dentures polymeric structure; (d) fractures of the connectors; (e) chipping of the aesthetic material of the fixed partial dentures and (f) comfort of the patients. The type of the opposite dentition (natural teeth, fixed partial denture, removable/complete denture) was also recorded.

Periodontal assessment was realized by clinical examination and intraoral radiographic examination. Fractures of RPD’s polymeric structure, fractures of the connectors, chipping of the aesthetic material of the fixed partial dentures were observed during clinical examination.

Maintenance of the occlusal contacts were checked by using 40µ articulation paper (Bausch – Germany). Masticatory efficiency was assessed by the administration of a questionnaire (including yes/no questions). The overall satisfaction of the patients was registered on a Likert scale 1 to 5: 1-not satisfied, 2-nearly satisfied, 3 satisfied, 4-very satisfied, 5-exceptional. All the check-ups were realized by two experienced clinicians. The initial periodontal therapy, scaling, professional cleaning and rinsing with chlorhexidine (0.06%%) and peroxide mouthwash (1.5%) was realized in all the cases. After that, the Oral Health Impact Profile-14 (OHIP-14) questionnaire was used to assess the quality of life of the RPD’s wearers included in this study.

**Fig. 1:**
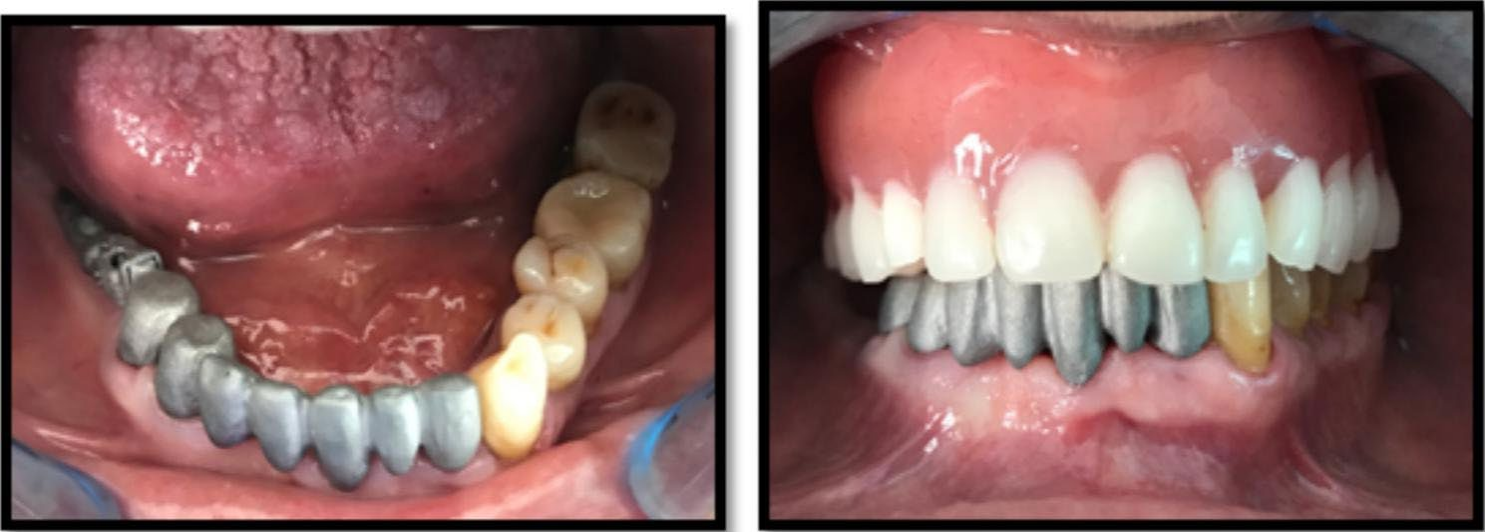
a and b. Try-in of the SLS metal framework (FPD and u-RPD)

The patients receiving u-RPD’s were treated following the next steps: preparation of the abutments for the FPD with external precision attachment. In patients who received a u-RPD and had before a hybrid treatment with bi-RPDs, the old FPD’s were removed. In the new cases, the abutment teeth were prepared for the FPDs very conservative, by using diamond burs (*Strauss&Co*, Germany) and a vertical prepared finishing line. After 10 days with provisional FPD’s (Protemp Plus-3 M Espe AG, Germany), the prepared abutment teeth were impressed by using a vinylpolysiloxane impression material in one step technique (Variotime easy putty and light flow, Kulzer, Germany). The impression of the antagonists was taken with the same material in one step and the occlusion was recorded by using a bite impression material (Occlufast, Zermack, Italy). Then, the try-in of the selective laser sintered (SLS) metal framework was performed (Fig. 1).

**Fig. 2:**
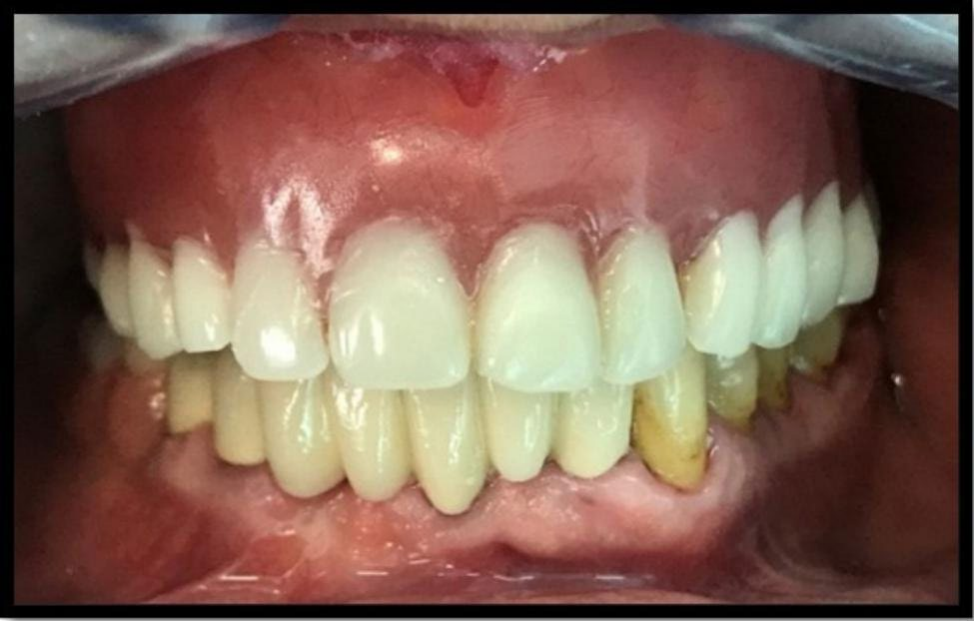
Intraoral aspect of the treatment before luting the FPD with u-RPD

The final FPD with the external precision attachment was received from the laboratory and a final impression for the RPD was taken by using polyether material (Impregum, 3 M Espe Germany) in an individual tray fabricated over the FPD. The occlusion registration was realized with occlusal rims. Maximum two artificial teeth were selected when the FPD was fixed on two mesial abutments. Consecutive. the try-in of the u-RPD and FPD was realized. In the final, the FPD was cemented together with the u-RPD by using a glass ionomer cement (Fuji Plus – GC Japan). The occlusal relationships were adjusted before and after the luting, by using 40µ and 10 µ microns thin articulating papers (Bausch – Germany) (Fig. 2).

The treatment of the patients with bi-RPD with a major connector (control group) followed the same clinical steps as in the u-RPD treatments. The same materials and instruments were used during the treatment. After the prosthetic treatment has been completed, the patients were instructed: (1) how to insert and remove the RPD; (2) to remove the RPD during night and keep it in a mug with water; (3) how to clean the RPD’s by using cleaning tabs and a dental brush and toothpaste; (4) to come for the recalls every 6 month and to call any time they had a complaint.

### Oral health questionnaire

The short form of the OHIP-14 questionnaire [22, 23] was used to evaluate the oral health of the patients after each type of administrated treatment. The questionnaire is organized into seven dimensions with two questions each: functional limitation, physical pain, psychological discomfort, physical disability, psychological disability, social disability, handicap, and addresses various aspects of oral health [23]. Each removable denture wearer responded to the 14 questions of the questionnaire. Each question had assigned a 5-point Likert scale (from 1 = never to 5 = very often) based answer. The total score was calculated by summing up all the points of the answered questions, and its range was from 0 to 56. A higher score indicated a worse impact on oral health.

### Statistical analysis

The statistical analysis was performed using SPSS version 25.0 (SPSS Inc., Chicago, IL) and R project packages for statistical computing. Descriptive statistics were presented as mean ± standard deviation for continuous variables or counts with percentage for the categorical ones.

Kaplan–Meier survival analysis was conducted using two criteria of failure as endpoints, i.e., when either a repair was needed or a complete failure occurred, discriminated according to RPD type. In addition, restorations not meeting the failure criterion during the five-year follow-up period were labeled as “censored”. A *p*-value of 0.05 was the threshold for the statistical significance.

## Results

The patients with u-RPD presented a mean age of 53.59 (± 11.41) years, 95% CI (47.72; 59.46), while the mean age of patients with bi-RPD was 57.94 (± 13.94) years, 95% CI (50.76; 65.12). The data sample’s characteristics are presented in Table 1.

**Table 1:** General characteristics of the patients with u-RPD vs. patients with bi-RPD

Variable	u-RPD	bi-RPD	p-value^(2)^
Age	53.59 ± 11.41^(1)^	57.94 ± 13.94^(1)^	0.454
Gender			
Men	6 (35.3%)	6 (35.3%)	NA
Women	11 (64.7%)	11 (64.7%)	NA
Jaw			
Mandibula	13 (76.5%)	13 (76.5%)	NA
Maxilla	4 (23.5%)	4 (23.5%)	NA
Kennedy class			0.006
Class I	2 (11.8%)	11 (64.7%)	
Class II	13 (76.5%)	5 (29.4%)	
Class III	2 (11.8%)	1 (5.9%)	
Satisfaction score	4.88 ± 0.48^(1)^	4.41 ± 0.62^(1)^	0.026

^(1)^ mean ± st.dev

^(2)^Mann-Whitney U test or Pearson chi-square test

Abbreviations: RPD, retained partial dentures

We described the RPDs of both groups of patients in Table 2.

**Table 2:** Characteristics of the RPDs

Type of RPD	Opposite denture					Denture type	
	Natural	Natural + FPD	FPD / FPD + RPD	Over-denture	Complete denture	FPD + RPD Rhein	FPD + RPD attachment
Unilateral	4 (23.5%)	4 (23.5%)	4 (23.5%)	2 (11.8%)	3 (17.6%)	14 (82.4%)	3 (17.6%)
Bilateral	3 (17.6%)	0 (0%)	12 (70.6%)	2 (11.8%)	0 (0%)	0 (0%)	17 (100%)

Abbreviations: RPD, retained partial dentures; FPD, fixed partial denture

We observed no significant differences between the general characteristics of the two groups of patients, i.e., both groups included 35.3% of men and 64.7% of women, both groups had the treatment on mandibula in proportion of 76.5% and maxilla in proportion of 23.3%. The group of patients receiving u-RPD presented Kennedy class I in proportion of 11.8%, while the patients receiving bi-RPD presented the same class in a significantly higher proportion 64.7% (Pearson chi-square test *X*^2^(1) = 10.091, *p* = 0.001). On the contrary, the group of patients receiving u-RPD presented Kennedy class II in proportion of 76.5%, while the patients receiving bi-RPD presented the same class in a significantly lower proportion 29.4% (Pearson chi-square test *X*^2^(1) = 7.556, *p* = 0.005). A small percent of patients presented Kennedy class III in both groups.

When comparing the oral health of patients with u-RPD vs. patients with bi-RPD, we observed that patients with u-RPD’s presented significantly better oral health in all OHIP-14 domains (Table 3).

**Table 3:** Comparison of the OHIP-14 scores between u-RPD and bi-RPD wearers

Domain^(1)^	u-RPD	bi-RPD	*p*-value^(2)^
Functional limitations	2.00 (2.00–2.00)	3.00 (2.00–4.00)	< 0.001
Physical pain	2.50 (2.00–3.00)	4.50 (4.00–5.00)	< 0.001
Psychological discomfort	2.00 (2.00–3.00)	4.00 (3.00–4.00)	< 0.001
Physical disability	2.00 (2.00-3.67)	3.00 (3.00–4.00)	< 0.001
Psychological disability	2.00 (2.00–4.00)	4.00 (3.00–4.00)	< 0.001
Social disability	2.00 (2.00–2.00)	3.00 (3.00–4.00)	< 0.001
Total score	15.00 (14.00–17.00)	23.00 (22.00–26.00)	< 0.001

^(1)^ median (inter-quartile range)

^(2)^ Mann-Whitney U test

Abbreviations: RPD, retained partial dentures

We conducted a survival analysis of the two types of RPD, namely, u-RPD and bi-RPD. The mean survival time in years was 4.882 ± 0.114, 95% CI (4.659; 5.106) and 4.882 ± 0.078, 95% CI (4.729; 5.036), for the u-RPD and the bi-RPD, respectively (Table 4).

**Table 4:** Results of the survival analysis

Type of RPD	Survival time [years] 95% CI	Survival rate [%]	Log-rank test
u-RPD	4.882 ± 0.114^(1)^ (4.659; 5.106)	94.1 ± 0.57^(2)^	*p* = 0.584
bi-RPD	4.882 ± 0.078^(1)^ (4.729; 5.036)	88.2 ± 0.78^(2)^	

^(1)^ mean ± st.dev

^(2)^ percent ± st.error

Abbreviations: RPD, retained partial dentures

**Fig. 3:**
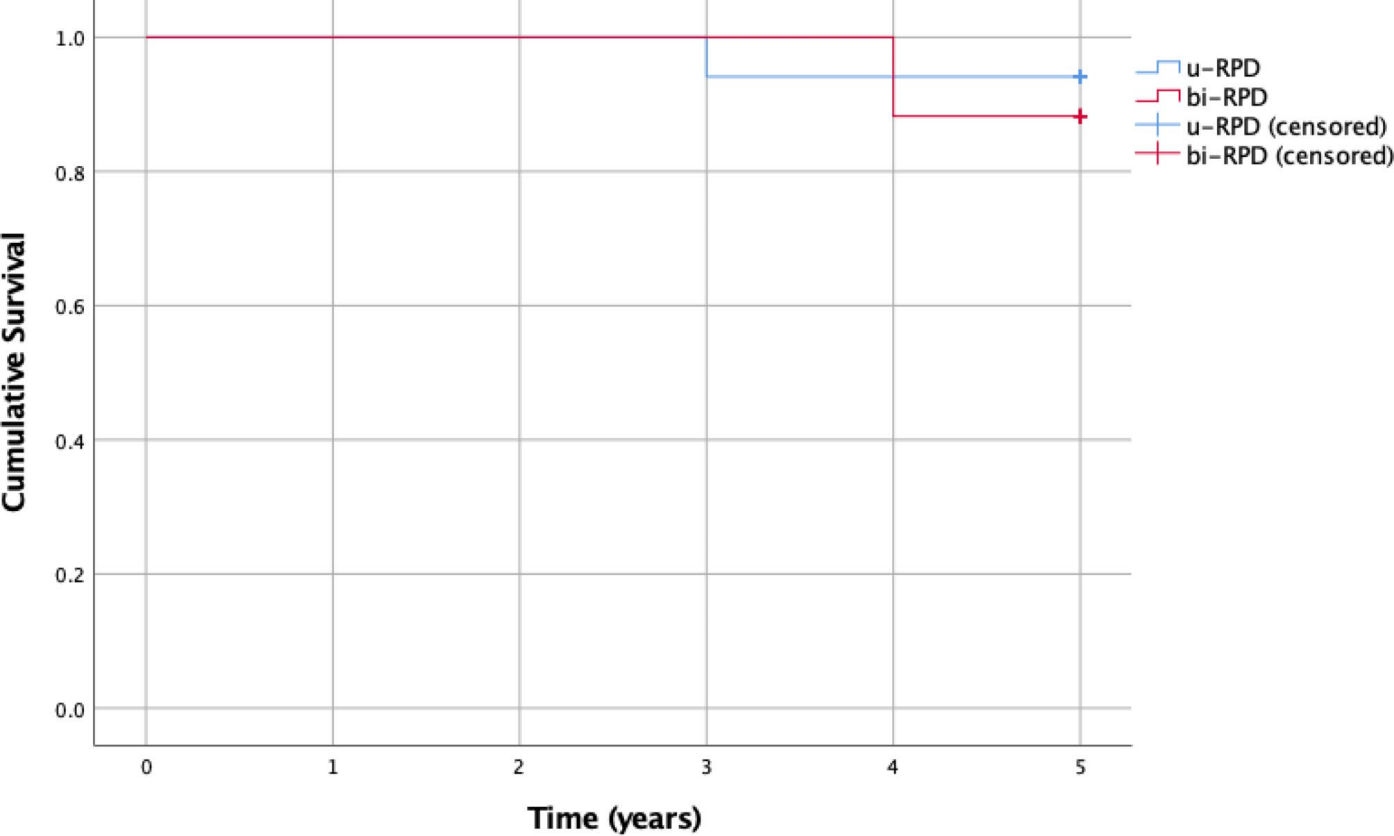
The Kaplan Mayer survival functions

The Kaplan–Meier five-year survival curves are shown in Fig. 3.

The five-year survival rates for the two dentures were 94.1% for u-RPD vs. 88.2% for bi-RPD with a major connector, without a statistically significant difference between them (Log-rank test χ^2^(1) = 0.301, *p* = 0.584).

The patients receiving u-RPD presented significantly higher satisfaction scores compared to the patients receiving bi-RPD, 4.88 ± 0.48 vs. 4.41 ± 0.62, Mann-Whitney U test, p = 0.026.

## Discussion

Improvements in OHQoL following provision of RPDs were still not consistently reported [24]. It was observed that RPD wearers still had impaired OHQoL, despite being rehabilitated [25–27]. So, we studied the overall satisfaction and OHQoL in RPD’s wearers: u-RPD’s versus bi-RPD’s. The present research has found that patients wearing RPD’s have a good OHQoL, but it depends on the type of RPD’s (u-RPD’s or bi-RPD’s). We observed that OHQoL was better in case of u-RPD’s wearers. Recent research [28] found also that unilateral nonmetal clasp dentures were better than conventional removable partial dentures for the OHQoL of individuals with unilateral distal-extension tooth loss in the mandible.

It was observed that masticatory performance compared with shortened dental arch improved with RPD, mainly among the younger age groups, in unilateral free end saddle male subjects [29–31]. In the present study, the maximum satisfaction was obtained in patients wearing before a bi-RPD, transformed into a resilient u-RPD’s (45%). It has been seen an improvement regarding the mastication in patients with dental shortened arches after treating them with u-RPD’s.

The survival of the u-RPD’s compared with bi- RPD’s were compared during the present study. There were no significant differences between the u-RPD’s survival (94.1%) and the bi-RPD’s (84.2%). According to studies recently published, similar survival rates (90- 94.7%) during a 10-year follow-up period were found [32, 33].

The bi-RPD’s were previously compared to u-RPD’s [34] and the conclusion was that u-RPD cannot be used as long-time prosthetic treatment. Other researcher’s conclusion states that if the situation demands, the possibility of unconventional designs such as modified spring cantilever or precision attachments should be well explored and adopted, but a highly skilled dental team and a specific patient presentation is required for them to be a reasonable and predictable prosthetic option [35, 36]. The present study’s results are in accordance with this last conclusion.

After comprehensive consideration, it is appropriate to choose two abutment teeth for restoration [37]. To protect the periodontal health of the abutment when restored with distal extension extra-coronal attachment dentures, it is necessary to examine periodically after restored, to keep the periodontal health of the abutment. In the present study, in all the cases treated with RPD’s at least two mesial abutments teeth were used. In two cases treated with u-RPD’s, where a distal abutment was present (class III Kennedy), it was used, and the support, retention, and stabilization were improved. Better scores in survival rates were obtained in these two cases.

A recent, very interesting three-dimensional finite element analysis [38] compared the effect of position of a distal implant abutment, in terminal edentate areas. A further clinical study regarding the comfort of the senior patients due to the improvement of u-PRD’s biodynamics would be of great interest.

Ending the denture teeth at the mesial cusp of second molar and positioning the occlusal contacts over the ridge crest adequately stabilize the abutment tooth and denture base of u-RPD [39]. All the dentures included in this study were manufactured according to this concept.

Another problem mentioned in the literature is the unnoticed swallow of the u-RPD [40], but also of the removable denture with major connector [41, 42]. Denture ingestion is more common among patients with psycho-neurologic deficit, alcohol, or drug abusers. Among healthy and younger population, denture ingestion is rare. Patients with denture loosening should be recommended to visit dentist as soon as possible [43]. One of the inclusion criteria in this study was that the patients must have good neuromotor reflexes, and they should come to check-ups every six month or every time when they noticed a dental problem. A better communication with the seniors was kept in constant telephone contact with them or their family.

Any prosthodontist is aware that treating seniors with removable dentures is a real challenge. Excellent communication with the patient and his family was extremely important during and following the prosthodontic treatment. Further clinical studies on many patients, comparing the u-RPD with and without a distal abutment (natural teeth or implant), using the new technologies would be interesting for restoring seniors’ dentition and for assuring them a better life comfort. The limitations of this study were the small number of patients included, and the comparisons between only two groups of removable denture wearers.

## Conclusion

Patients receiving u-RPD presented higher levels of treatment satisfaction and better oral health than patients receiving bi-RPD. The survival rates of the treatments u-RPD and bi-RPD were similar.

## Data Availability

The datasets used and/or analysed during the current study available from the corresponding author on reasonable request.
